# The mediating role of subjective social status in the association between objective socioeconomic status and mental health status: evidence from Iranian national data

**DOI:** 10.3389/fpsyt.2024.1427993

**Published:** 2024-09-30

**Authors:** Nastaran Nasirpour, Kasra Jafari, Mojtaba Habibi Asgarabad, Masoud Salehi, Masoumeh Amin-Esmaeili, Afarin Rahimi-Movaghar, Seyed Abbas Motevalian

**Affiliations:** ^1^ Department of Epidemiology, School of Public Health, Iran University of Medical Sciences, Tehran, Iran; ^2^ Research Center for Addiction and Risky Behaviors (ReCARB), Psychosocial Health Research Institute, Iran University of Medical Sciences, Tehran, Iran; ^3^ Department of Psychology, Norwegian University of Science and Technology, Trondheim, Norway; ^4^ Department of Biostatistics, School of Public Health, Iran University of Medical Sciences, Tehran, Iran; ^5^ Department of Mental Health, Johns Hopkins Bloomberg School of Public Health, Baltimore, MD, United States; ^6^ Iranian National Center for Addictions Studies, Tehran University of Medical Sciences, Tehran, Iran

**Keywords:** objective socioeconomic status, subjective social status, mental health, mediation analysis, household survey

## Abstract

**Introduction:**

Low socioeconomic status (SES) is identified as a pivotal risk factor for mental health. Objective socioeconomic status (OSS) is measured by tangible resources, whereas subjective social status (SSS) reflects an individual’s perception of their OSS. A paucity of literature exists that elucidates the specific psychosocial mechanisms or pathways linking OSS with mental illness via SSS. This research aimed to explore the mediating role of SSS in the OSS-mental health association, utilizing data from the Iranian Mental Health Survey (IranMHS).

**Methods:**

This study is a secondary analysis of data from IranMHS, a national survey conducted in 2011 through face-to-face interviews with 7,886 Iranian adults aged 15-64. As part of the original survey, the General Health Questionnaire-28 (GHQ-28) was randomly administered to half of these participants. We focused our analysis on data from 3,759 participants who completed all items of the GHQ-28. OSS was evaluated using education, occupation, wealth, and a combined OSS index, while SSS was measured using the MacArthur Scale. We examined how SSS mediates the associations between OSS factors and GHQ scores, including its four dimensions—somatic symptoms, anxiety and insomnia, social dysfunction, and severe depression— while adjusting for demographic variables and conducting gender-stratified analyses.

**Results:**

Among the 3759 respondents, 2157 (57.4%) were women, 2110 (56.1%) lived in urban areas, with an average age of 33.1 years (SD=12.1). SSS mediated the associations between wealth and the overall GHQ score (mediation percentage: 92.3%), education (75.4%), OSS index (66.7%), and occupation (34.0%) on the GHQ score. The most significant mediation effects were observed for wealth on the somatic symptoms, social dysfunction, and depression dimensions, with SSS accounting for more than 80% of these associations.

**Conclusion:**

The findings indicate a significant mediating role of SSS in the relationship between OSS and mental health. Enhancing our comprehension of the social determinants that moderate the relationship between objective and subjective socioeconomic status may contribute to a more nuanced understanding of the impact of SES on mental health outcomes.

## 1 Introduction

Mental disorders continue to be one of the top ten leading causes of burden worldwide, elucidating 16% of global disability-adjusted life years (DALYs) (1). Mental health is not solely the absence of mental disorders but also encompasses mental well-being, including the realization of one’s abilities, the capability to cope with life’s stresses, the capacity to work productively and fruitfully, and the contribution to one’s community (2). A substantial proportion (75%) of those afflicted by mental health disorders reside within the confines of low- and lower-income nations (3). A national survey conducted in Iran, a lower-middle-income country, found that the prevalence of mental health issues was 29.7%, with a significantly higher prevalence observed in women (31.8%) compared to men (26.6%) based on the General Health Questionnaire-28 (GHQ-28) (4).

The influence of improved objective socioeconomic status (OSS) on enhancing mental health has been increasingly demonstrated (5). A recent review indicated that individuals maintaining consistently high OSS exhibit the most favorable mental health, followed by those who experience upward social mobility (5). Moreover, a population-based survey conducted in Tehran, Iran, indicated low OSS was associated with poor mental health, as measured by the GHQ-28 (6). The study further highlighted that among the four dimensions of the GHQ-28, depression and anxiety emerged as the most influential determinants of mental health (6). OSS, encompassing tangible resources, is frequently assessed through education, occupation, and income (7). Established mechanisms such as coping strategies, resilience, access to healthcare resources, and life stressors have been recognized in the relationship between OSS and mental health (5), yet the role of subjective social status has received comparatively less scholarly attention. There is evidence that the perceived value of OSS possessions partially underlies mental health disparities (8).

The construct of subjective social status (SSS) refers to an individual’s self-evaluation of their OSS within the social hierarchy, while simultaneously being influenced by psychological attributes (9). Results from the international mental health surveys have found an inverse association between SSS and mental illnesses after adjusting for OSS indicators in low-, middle-, and high-income countries (10). A population-based study in Tehran found that the average SSS score, measured by MacArthur’s scale, among 1,000 individuals, was 3.3 (11). SSS emerged as a significant indicator of self-assessed health (11).

The social rank theory posits that perceiving oneself as having a lower social status induces feelings of inadequacy and chronic stress, which adversely affect mental health (12). Additionally, the relationship between SSS and subjective well-being (SWB) can be understood through Antonovsky’s salutogenic theory, which suggests that a strong sense of coherence can mitigate the stressors associated with low OSS (13). The established pathways among OSS-mental health, OSS-SSS, and SSS-mental health provide a hypothetical causal chain, in which an independent variable (OSS) is hypothesized to influence a mediating variable (SSS), which in turn is associated with a dependent variable (mental health status). However, only two cross-sectional studies (7, 14), and one national longitudinal study (15), have investigated the indirect association between objective SES (OSS) and mental health via subjective SES (SSS), according to our knowledge. The findings of a national health survey in Germany suggest that SSS acts as a mediator in the relationship between common indicators of OSS with depressive symptoms (7). Similarly, data from the English context indicated that SSS partially or fully acted as a mediating factor in the relationship between education, occupation, and wealth with self-reported depression (14).

This relationship can be further elucidated through the lens of mindsponge theory, which posits that information (such as OSS) is processed through multiple filters grounded in core values, leading to the formation of perceptions (such as SSS) (16). The social identity theory also explains this indirect effect through SSS, whereby subjective class identity refers to an individual’s perception of their standing within the social class hierarchy, particularly as influenced by OSS (17). Lower perception of social status (SSS) may result in adverse emotional reactions such as frustration, shame, inferiority, stress, and a pessimistic outlook, ultimately leading to the development of mental disorders (15). The experiential manipulation of people’s subjective SES can lead to changes in their behaviors and abilities, ultimately affecting their mental well-being (18).

There are documented variations in the social factors influencing mental health by gender. A lifetime follow-up study in the Basque Country revealed that women from low-income households exhibited higher prevalences of depression and anxiety compared to their male counterparts in similar economic circumstances (19). Furthermore, a representative sample from the Korean Longitudinal Study of Aging, indicates that educational attainment functions as a significant protective factor against depressive symptoms only in women (20). Additionally, recent research conducted in Iran has demonstrated that gender had a significant contribution to the difference in mental health between high and low-wealth groups (21). Moreover, numerous studies have shown that the impact of SSS on depressive symptoms is significantly more pronounced among women populations (22, 23).

The current study aimed to investigate the mediating role of SSS in the relationship between education, occupation, wealth, and a composite measure of them (OSS index), with mental health status separately by gender among participants of the 2011 Iranian National Mental Health Survey (IranMHS). The high prevalence of mental health problems identified in the 2011 IranMHS underscores an urgent need for targeted resources and interventions (24), a need likely exacerbated by current socioeconomic status (SES) disparities (4, 6). This research investigated the mechanisms through which various OSS indicators influence mental health status via the mediating role of SSS, using a nationally representative sample and considering gender stratification. Additionally, this study uniquely examines the indirect associations of OSS with four specific dimensions of mental health status: somatic symptoms, anxiety and insomnia, social dysfunction, and depression.

## 2 Materials and methods

### 2.1 Study design and participants

The current study is a secondary analysis of data from the Iran Mental Health Survey (IranMHS), a cross-sectional national household survey conducted between January and June 2011. The primary aim of the IranMHS was to assess the 12-month prevalence and severity of mental disorders among the adult population in Iran. The survey employed a three-stage probability sampling method to select a representative sample of Iranian household residents aged 15 to 64 years. In the first stage, 1,525 blocks were selected across all provinces of Iran, with the number of blocks in each province determined proportionally to its population size. These blocks, the smallest geographic units defined by public paths or natural structures, were drawn from a national list based on the 2006 census. In the second stage, six households were selected from each block using a systematic random sampling method. In the third stage, all residents in the selected households were listed through interviews with a household informant, with inclusion and exclusion criteria applied to each listed resident. The Kish grid method was then used to randomly select one eligible individual from each household for participation in the study (24).

The survey achieved a response rate of 86.2%, resulting in a final sample of 7,886 participants who completed various diagnostic interviews and questionnaires. As part of the original IranMHS study, a randomly selected half of the participants (3,759 individuals) were administered the General Health Questionnaire-28 (GHQ-28) at the time of their interview (24). This was done to facilitate comparisons with previous studies that had also used the GHQ-28. The present study exclusively utilizes data from the 2011 IranMHS, focusing on sociodemographic variables, objective socioeconomic status (OSS) factors (including education, occupation, and wealth), subjective social status (SSS), and the GHQ-28 results.

### 2.2 Measurements

#### 2.2.1 Objective socioeconomic status

The OSS is the social and economic position of an individual in relation to others, which is typically measured by their educational attainment, occupation, income, and wealth. In this study, we categorized education into six levels based on the highest level completed, including illiterate, primary school, middle school, high school, high school diploma, and any university education. The occupational status was considered as an ordinal variable with the following six levels: unemployed, homemaker, retired, student, part-time, and full-time employment. In the IranMHS, the measurement of wealth was derived from a self-reported inventory of household assets and amenities. To estimate wealth levels, we first conducted a principal component analysis (PCA) on household assets to derive a quantitative wealth index (25). For details see **Supplementary Table S1**. We divided the individuals into six equal groups based on their wealth index score, with the sixth group having the highest wealth. Details of ranges for wealth groups are presented in **Supplementary Table S2**. By summation of three six-level variables of education, occupational status, and wealth; we calculated the “OSS index,” which ranged from 3 to 18. Subsequently, we categorized the index into four equal groups and assigned the highest status to the fourth group.

#### 2.2.2 Subjective social status

Subjective SES states a person’s self-perceived position in the social order. To measure SSS, MacArthur’s subjective social status scale, as developed by Adler, which involves using a picture of a 10-rung ladder was employed. The top rung demonstrates the highest SES, high educational attainment, prestigious occupation, and high income, and the bottom rung signifies the lowest SES, involving the minimum education, low status or menial occupation, and low income (26). Participants were requested to position themselves on the ladder relative to others in their society (26). This scale demonstrated adequate reliability (27, 28), and has been employed in extensive epidemiological investigations in Europe, the United States as well as Iran (11, 27). Moreover, during the pilot stage of the IranMHS, the interrater reliability of the SSS was evaluated (24), and the kappa coefficient was determined to be 0.75, which we present this result for the first time in the current study. We stratified the decile-ranked SSS variable into four distinct categories and conferred the highest status to the fourth group.

#### 2.2.3 Outcome measure

The Persian version of the GHQ-28 was utilized to assess mental health status. The GHQ-28 was developed as a screening tool to differentiate psychiatric patients from healthy individuals. The structure of the GHQ-28 involves four subscales: somatic symptoms (A scale, A1-A7), anxiety and insomnia (B scale, B1-B7), social dysfunction (C scale, C1-C7), and severe depression (D scale, D1-D7). This is a self-administered screening questionnaire asking participants to evaluate their recent distressing symptoms over the past 30 days (29). We employed a Likert scale of 0 to 3 for scoring the questions, with the following ordinal categories: 0 (never), 1 (as usual), 2 (almost more than usual), and 3 (more than usual), yielding a total score of zero to 84 (29). The scoring range for each dimension is from 0 to 21 inclusive (29). The standardized Persian version of GHQ-28 demonstrated high reliability and validity, with a reported sensitivity of 84.7%, a specificity of 93.8%, and an overall misclassification rate of 8.2% (30).

#### 2.2.4 Covariates

**Figure 1:**
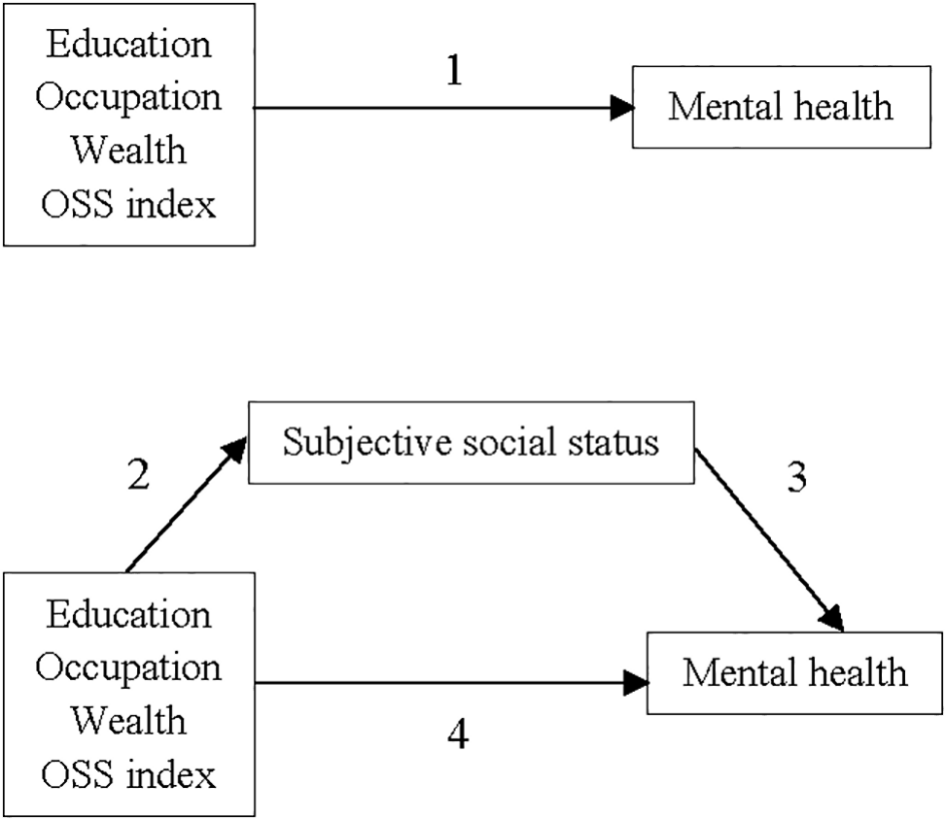
Mediating pathway of the association between objective socioeconomic status (education, occupation, wealth, and OSS index) and mental health status by subjective social status.

We selected a set of potential control variables that may be associated with dependent variables (**Figure 1**, paths 1, 2, and 3) (31). In gender subgroups, we considered age, place of residence, and marital status. In the total sample, we selected the same confounding variables as well as gender. Regarding age, we grouped individuals into six groups. In the context of gender identification, the binary category of men and women is used. For the place of residence, we considered urban and rural areas, and for marital status, we categorized it into three groups, married, never married, and previously married (divorced or widowed).

### 2.3 Ethical statement

The study was approved by the research ethics committee of the Iran University of Medical Science with ethical code #IR.IUMS.REC.1402.1032. An informed consent had been obtained from all the study participants before engaging in the IranMHS. Moreover, the participants’ data were kept confidential and only available to the primary researchers of the study. Anonymized data was utilized for statistical analyses. We conducted the study following the Declaration of Helsinki (32), national guidelines, and regulations.

### 2.4 Statistical analyses

Descriptive statistics were presented as the mean and standard error (SE) for continuous variables and as numbers (percentages) for categorical variables. All analyses were conducted independently for men and women. In the analyses of gender subgroups, we adjusted for age, place of residence, and marital status. In the analyses of the total sample, we controlled for the same confounding variables as well as gender. STATA version 14 was used for all statistical analyses, and the significance threshold was established at 0.05.

Survey weights for each individual were derived from the multiplicative combination of inverse probability of unit selection (w1), non-response adjustments (w2), and post-stratification adjustments (w3). All results are based on complex sample survey analysis, accounting for clusters and the calculated weights (24, 31).

We utilized linear regression analyses to investigate the associations between each of the OSS indicators (education, occupation, wealth, and OSS index), SSS, and GHQ score (**Figure 1**, paths 1, 2, and 3). For regression analyses, standardized values of education, occupation, wealth, OSS index, and SSS were used. We calculated the Beta (β) of the dependent variables per 1 standard deviation increase in each of the independent variables. Two models were used in each linear regression analysis: no adjustment, and full adjustment for demographic confounders.

We required a mediation model to examine the mediating role of the SSS score in the relationship between OSS indicators scores and GHQ score as well as its four dimensions scores, adjusting for demographic confounders. The output of mediation analysis is presented in terms of total effect, direct effect, and indirect effect. The direct effect denotes the influence of an independent variable on a dependent variable while maintaining the mediator as a constant. This effect signifies the relationship that would be discerned if the mediator were stabilized at a specific value. The indirect effect, on the other hand, represents the influence of an independent variable on a dependent variable transmission through the mediator. When there are both direct and indirect effects, it is referred to as complementary mediation (partial mediation), while when the indirect effect is significant, but not the direct effect, it is referred to as indirect-only mediation (full mediation) (33). We utilized the KHB (Karlson, Holm, and Breen) command in STATA, a user-defined function for conducting mediation analyses (34). It is based on the work of Baron and Kenny, who developed a widely used method for mediation analysis (35, 36). The KHB method is primarily designed for various variants of logit models, but it can also be applied to linear regression (36). We also calculated the percentage of mediation by SSS as the ratio of the indirect association to the total association.

## 3 Results

Out of 3,759 study participants, 2,157 (57.4%) were women, 2,110 (56.1%) were urban, and the mean (SD) age was 33.1 (12.1) years. It was observed women exhibited a higher mean GHQ score compared to men (p-value < 0.001). **Table 1** presents the GHQ score by demographic characteristics of men and women IranMHS participants. Individuals with higher levels of OSS index and SSS exhibited lower mean values for GHQ.

**Table 1:** GHQ score in the IranMHS participants by demographic characteristics.

	Total			Men		Women	
	N	Mean (SE)	P-value	N	Mean (SE)	N	Mean (SE)
Overall	3759	17.6 (0.2)		1602	16.1 (0.3)	2157	19.2 (0.3)
Age							
15-19	481	16.6 (0.6)	< 0.01^*^	220	14.2 (0.7)	261	19.2 (0.9)
20-29	1212	17.4 (0.3)		495	16.4 (0.5)	717	18.4 (0.5)
30-39	1065	17.8 (0.4)		466	16.9 (0.6)	599	18.8 (0.5)
40-49	522	18.8 (0.6)		239	17.3 (0.8)	283	20.3 (0.8)
50-59	354	18.3 (0.7)		141	14.8 (0.7)	213	21.4 (1.0)
60-64	125	17.7 (0.9)		41	18.0 (1.6)	84	17.5 (1.0)
Residence							
Urban	2110	17.7 (0.3)	< 0.01^*^	900	16.3 (0.4)	1210	19.2 (0.4)
Rural	1649	17.4 (0.4)		702	15.7 (0.4)	947	19.1 (0.5)
Marital status							
Never married	979	17.2 (0.4)	< 0.05^*^	511	16.0 (0.5)	468	19.1 (0.7)
Married	2614	17.7 (0.3)		1064	16.1 (0.3)	1550	19.1 (0.3)
Previously married	166	20.8 (1.1)		27	21.9 (2.9)	139	20.5 (1.1)
Education							
Illiterate	314	20.4 (0.9)	< 0.001^*^	54	17.4 (1.7)	260	21.1 (1.0)
Primary school	891	19.3 (0.5)		329	17.6 (0.7)	562	20.5 (0.6)
Middle school	615	17.7 (0.5)		316	16.4 (0.7)	299	19.5 (0.7)
High school	619	16.4 (0.5)		319	14.5 (0.6)	300	19.0 (0.8)
High school diploma	711	17.3 (0.5)		309	16.4 (0.7)	402	18.2 (0.6)
University	599	16.7 (0.5)		269	16.1 (0.7)	330	17.4 (0.6)
Occupation							
Unemployed	345	19.6 (0.8)	< 0.001^*^	229	19.1 (0.9)	116	20.8 (1.4)
Homemaker	1546	19.4 (0.4)		9	29.8 (10.1)	1537	19.3 (0.3)
Retired	77	15.5 (0.9)		49	15.2 (1.1)	28	16.0 (1.2)
Student	444	16.5 (0.6)		203	14.1 (0.6)	241	19.1 (0.9)
Part-time	508	16.9 (0.5)		462	16.6 (0.6)	46	20.4 (2.0)
Full-time	839	16.0 (0.4)		650	15.6 (0.4)	189	17.6 (0.9)
Wealth							
1	614	19.6 (0.6)	< 0.001^*^	243	17.8 (0.9)	371	21.2 (0.8)
2	590	18.8 (0.6)		244	18.1 (1.0)	346	19.5 (0.7)
3	604	17.5 (0.5)		258	15.9 (0.6)	346	19.1 (0.8)
4	611	17.9 (0.5)		243	15.6 (0.7)	368	19.9 (0.7)
5	602	16.8 (0.4)		263	15.7 (0.6)	339	17.8 (0.6)
6	648	16.7 (0.4)		309	15.4 (0.6)	339	18.3 (0.7)
OSS index							
1 (3-7)	859	20.7 (0.5)	< 0.001^*^	143	19.2 (1.2)	716	21.1 (0.6)
2 (8-10)	990	18.3 (0.4)		371	17.8 (0.7)	619	18.7 (0.5)
3 (11-13)	930	17.0 (0.4)		473	15.4 (0.5)	457	18.8 (0.6)
4 (14-18)	880	16.2 (0.4)		567	15.5 (0.4)	313	17.6 (0.7)
SSS							
1 (1-2)	668	21.4 (0.6)	< 0.001^*^	330	19.6 (0.8)	338	23.9 (0.9)
2 (3-4)	1246	18.7 (0.4)		573	17.1 (0.5)	673	20.6 (0.6)
3 (5)	1383	16.1 (0.3)		524	14.1 (0.4)	859	17.7 (0.4)
4 (6-10)	455	15.5 (0.5)		175	14.5 (0.8)	280	16.5 (0.7)

^*^Significant.

### 3.1 SES indicators and GHQ

**Table 2** details the outcomes of multiple linear regression analyses examining the relationship between five SES indicators—education, occupation, wealth, OSS index, and SSS—and psychological distress, as measured by the GHQ score. The results demonstrate that higher levels of education, occupation, wealth, and OSS index are associated with lower GHQ score, indicating reduced psychological distress across participants. Gender-stratified analysis shows variations in the associations of education and occupation between men and women; however, these interactions are not statistically significant (p=0.12 for education and p=0.42 for occupation), suggesting that while educational attainment more strongly affects women, occupational status more strongly affects men, these differences are not statistically robust. SSS emerged as the most impactful SES indicator, significantly associated with lower GHQ score in both the overall and gender-specific analyses.

**Table 2:** Linear regression results of SES indicators and GHQ score in the IranMHS.

	Model 1	Model 2
	β (95% CI)	β (95% CI)
Total population		
Education	-0.65 (-0.92- -0.38)^**^	-0.58 (-0.86- -0.30)^**^
Occupation	-0.80 (-1.02- -0.58)^**^	-0.48 (-0.77- -0.20)^*^
Wealth	-0.57 (-0.81- -0.32)^**^	-0.72 (-1.00- -0.45)^**^
OSS index	-0.43 (-0.54- -0.32)^**^	-0.40 (-0.54- -0.27)^**^
SSS	-1.02 (-1.25- -0.79)^**^	-1.17 (-1.40- -0.93)^**^
Men		
Education	-0.29 (-0.68-0.10)	-0.29 (-0.68-0.11)
Occupation	-0.57 (-0.95- -0.19)^*^	-0.62 (-1.03- -0.21)^*^
Wealth	-0.52 (-0.87- -0.18)^*^	-0.75 (-1.15- -0.36)^**^
OSS index	-0.33 (-0.50- -0.16)^**^	-0.42 (-0.60- -0.23)^**^
SSS	-1.00 (-1.34- -0.67)^**^	-1.11 (-1.45- -0.76)^**^
Women		
Education	-0.76 (-1.10- -0.41)^**^	-0.87 (-1.25- -0.48)^**^
Occupation	-0.37 (-0.77-0.04)	-0.38 (-0.80-0.05)
Wealth	-0.53 (-0.87- -0.19)^*^	-0.69 (-1.08- -0.31)^**^
OSS index	-0.33 (-0.50- -0.17)^**^	-0.41 (-0.60- -0.23)^**^
SSS	-1.17 (-1.48- -0.86)^**^	-1.24 (-1.56- -0.92)^**^

Model 1 unadjusted. Model 2 adjusted for age, residence, marital status, and gender (only in the total sample).

^*^Sig. p < 0.01.

^**^Sig. p < 0.001.

### 3.2 OSS indicators and SSS

**Table 3** presents the results of linear regression analyses assessing the associations between OSS indicators and SSS. Adjusted for demographic variables in Model 2, the data indicate that higher levels of education, occupation, wealth, and OSS index significantly enhance SSS score. Significantly stronger associations between SSS and education, wealth, and the OSS index were observed in men compared to women, with p-values for interactions at less than 0.01, 0.05, and 0.001, respectively. Wealth was identified as having the strongest link to SSS among the evaluated factors.

**Table 3:** Linear regression results of OSS indicators and SSS in the IranMHS.

	Model 1	Model 2
	β (95% CI)	β (95% CI)
Total population		
Education	0.42 (0.38-0.47)^*^	0.39 (0.34-0.43)^*^
Occupation	0.08 (0.05-0.12)^*^	0.14 (0.10-0.19)^*^
Wealth	0.54 (0.50-0.57)^*^	0.54 (0.49-0.58)^*^
OSS index	0.21 (0.20-0.23)^*^	0.24 (0.22-0.26)^*^
Men		
Education	0.51 (0.45-0.58)^*^	0.45 (0.37-0.52)^*^
Occupation	0.15 (0.08-0.21)^*^	0.16 (0.10-0.22)^*^
Wealth	0.58 (0.53-0.64)^*^	0.57 (0.51-0.63)^*^
OSS index	0.28 (0.26-0.31)^*^	0.27 (0.24-0.30)^*^
Women		
Education	0.38 (0.33-0.43)^*^	0.35 (0.29-0.41)^*^
Occupation	0.23 (0.16-0.29)^*^	0.15 (0.08-0.22)^*^
Wealth	0.50 (0.45-0.55)^*^	0.50 (0.44-0.55)^*^
OSS index	0.23 (0.20-0.25)^*^	0.22 (0.19-0.24)^*^

Model 1 unadjusted. Model 2 adjusted for age, residence, marital status, and gender (only in the total sample).

^*^Sig. p < 0.001.

### 3.3 Mediation of SSS in the association between OSS indicators and GHQ

**Table 4** illustrates the total (path 1), direct (path 4), and indirect (paths 2 and 3) associations between OSS factors and the GHQ score (**Figure 1**), along with the mediated proportions after controlling for covariates in the overall population and gender subgroups. The total association between each OSS factor and the GHQ score was statistically significant in the entire population as well as in both men and women.

**Table 4:** KHB test for association between OSS indicators and GHQ score mediated through SSS in the IranMHS.

	Total association^a^	Direct association^a^	Indirect association^a^	% Mediated^b^
	β (95% CI)	β (95% CI)	β (95% CI)	
Total population				
Education	-0.61 (-0.85- -037)^***^	-0.15 (-0.40-0.10)	-0.46 (-0.54- -0.37)^***^	75.4
Occupation	-0.50 (-0.72- -0.28)^***^	-0.33 (-0.55- -0.10)^**^	-0.17 (-0.23- -0.12)^***^	34.0
Wealth	-0.65 (-0.87- -0.44)^***^	-0.05 (-0.30-0.19)	-0.60 (-0.71- -0.49)^***^	92.3
OSS index	-0.39 (-0.50- -0.29)^***^	-0.14 (-0.25- -0.02)^*^	-0.26 (-0.31- -0.21)^***^	66.7
Men				
Education	-0.46 (-0.80- -0.11)^**^	0.06 (-0.30-0.43)	-0.52 (-0.67- -0.38)^***^	NA^c^
Occupation	-0.63 (-0.91- -0.34)^***^	-0.46 (-0.75- -0.17)^**^	-0.17 (-0.24- -0.10)^***^	27.0
Wealth	-0.69 (-0.99- -0.38)^***^	-0.10 (-0.44-0.25)	-0.59 (-0.76- -0.42)^***^	85.5
OSS index	-0.43 (-0.58- -0.28)^***^	-0.17 (-0.34-0.00)^*^	-0.26 (-0.34- -0.18)^***^	60.5
Women				
Education	-0.71 (-1.04- -0.37)^***^	-0.29 (-0.63-0.06)	-0.42 (-0.53- -0.30)^***^	59.2
Occupation	-0.39 (-0.75- -0.03)^*^	-0.19 (-0.55-0.17)	-0.20 (-0.28- -0.12)^***^	51.3
Wealth	-0.63 (-0.93- -0.32)^***^	-0.03 (-0.36-0.31)	-0.60 (-0.75- -0.45)^***^	95.2
OSS index	-0.37 (-0.52- -0.22)^***^	-0.11 (-0.27-0.06)	-0.26 (-0.33- -0.19)^***^	70.3

a Adjusted for age, residence, marital status, and gender (only in the total sample).

b % The mediated association was computed as the ratio of the indirect association to the total association.

c Full mediation

^*^Sig. p < 0.05.

^**^Sig. p < 0.01.

^***^Sig. p < 0.001.

NA, not applicable.

The analysis revealed a significant indirect association between education and reducing GHQ score, with 75.4% of this association mediated by SSS. The relationship between occupation and GHQ score was partially mediated by SSS, accounting for 34.0% of the association. Notably, the influence of wealth on GHQ score was fully mediated through SSS (92.3%), and SSS explained two-thirds of the total association of the OSS index (66.7%).

In gender-stratified analyses, the indirect associations between education and GHQ score through SSS were significant for both men (β: -0.52) and women (β: -0.42). Conversely, the direct association of education varied by gender, being slightly positive in men (β: 0.06) and notably negative in women (β: -0.29), with this gender difference reaching statistical significance (p-for-interaction < 0.05). Additionally, SSS significantly mediated the association between occupation and mental health status, with mediating proportions of 27.0% for men and 51.3% for women. Moreover, the total association of wealth was highly mediated by SSS both in men (85.5%) and in women (95.2%). Lastly, the indirect association of the OSS index through SSS was substantial, mediating 60.5% of the association in men and 70.3% in women.

The total association of each OSS factor, along with the indirect associations of these factors on the GHQ score do not exhibit a statistically significant difference between men and women participants.

**Table 5** presents the total and indirect associations of OSS factors with the scores across four dimensions of the GHQ-28, also detailing the mediation percentages after adjusting for covariates within the overall population and gender subgroups. Notably, the total associations between wealth and somatic symptoms, social dysfunction, as well as depression were substantially mediated by SSS, accounting for 87.1%, 81.0%, and 83.3%, respectively. Furthermore, the relationships between education, wealth, and the OSS index with anxiety and insomnia were fully mediated by SSS. Additionally, the total and indirect associations of each objective SES factor with the dimensions of the GHQ-28 did not exhibit statistically significant differences between men and women.

**Table 5:** KHB test for associations between OSS indicators and dimensions scores of GHQ-28 mediated by SSS in the IranMHS.

Exposure	Association	Somatic symptoms		Anxiety and insomnia		Social dysfunction		Depression	
		β (95% CI)^a^	% Mediated^b^	β (95% CI)^a^	% Mediated^b^	β (95% CI)^a^	% Mediated^b^	β (95% CI)^a^	% Mediated^b^
Total population									
Education	Total Indirect	-0.34 (-0.46- -0.22)^***^ -0.18 (-0.22- -0.14)^***^	52.9	-0.13 (-0.25-0.005) -0.21 (-0.25- -0.16)^***^	NA^c^	-0.25 (-0.33- -0.16)^***^ -0.12 (-0.15- -0.09)^***^	48.0	-0.26 (-0.38- -0.13)^***^ -0.22 (-0.27- -0.17)^***^	84.6
Occupation	Total Indirect	-0.27 (-0.40- -0.15)^***^ -0.08 (-0.11- -0.05)^***^	29.6	-0.17 (-0.31- -0.04)^**^ -0.08 (-0.11- -0.06)^***^	47.1	-0.22 (-0.31- -0.13)^***^ -0.05 (-0.07- -0.04)^***^	22.7	-0.24 (-0.37- -0.11)^***^ -0.09 (-0.12- -0.07)^***^	37.5
Wealth	Total Indirect	-0.31 (-0.43- -0.19)^***^ -0.27 (-0.33- -0.21)^***^	87.1	-0.24 (-0.36- -0.11)^***^ -0.29 (-0.36- -0.22)^***^	NA^c^	-0.21 (-0.30- -0.13)^***^ -0.17 (-0.21- -0.13)^***^	81.0	-0.36 (-0.48- -0.23)^***^ -0.30 (-0.36- -0.24)^***^	83.3
OSS index	Total Indirect	-0.44 (-0.57- -0.32)^***^ -0.24 (-0.30- -0.18)^***^	54.5	-0.26 (-0.40- -0.13)^***^ -0.28 (-0.35- -0.22)^***^	NA^c^	-0.34 (-0.43- -0.25)^***^ -0.15 (-0.19- -0.10)^***^	44.1	-0.42 (-0.55- -0.29)^***^ -0.29 (-0.35- -0.23)^***^	69.0
Men									
Education	Total Indirect	-0.24 (-0.41- -0.06)^**^ -0.17 (-0.24- -0.10)^***^	70.8	-0.03 (-0.23-0.17) -0.24 (-0.32- -0.16)^***^	NA^c^	-0.24 (-0.37- -0.11)^***^ -0.15 (-0.20- -0.10)^***^	62.5	-0.22 (-0.39- -0.04)^**^ -0.27 (-0.34- -0.19)^***^	NA^c^
Occupation	Total Indirect	-0.32 (-0.48- -0.15)^***^ -0.06 (-0.10- -0.03)^***^	18.8	-0.24 (-0.42- -0.05)^**^ -0.08 (-0.12- -0.04)^***^	33.3	-0.28 (-0.41- -0.16)^***^ -0.06 (-0.08- -0.03)^***^	21.4	-0.29 (-0.45- -0.12)^***^ -0.10 (-0.14- -0.06)^***^	34.5
Wealth	Total Indirect	-0.29 (-0.46- -0.13)^***^ -0.21 (-0.30- -0.12)^***^	72.4	-0.25 (-0.44- -0.06)^**^ -0.29 (-0.40- -0.19)^***^	NA^c^	-0.20 (-0.32- -0.07)^**^ -0.20 (-0.27- -0.14)^***^	NA^c^	-0.43 (-0.60- -0.27)^***^ -0.30 (-0.39- -0.21)^***^	69.8
OSS index	Total Indirect	-0.43 (-0.60- -0.25)^***^ -0.19 (-0.28- -0.10)^***^	44.2	-0.28 (-0.48- -0.07)^**^ -0.30 (-0.40- -0.19)^***^	NA^c^	-0.38 (-0.51- -0.25)^***^ -0.17 (-0.24- -0.10)^***^	44.7	-0.50 (-0.67- -0.32)^***^ -0.30 (-0.40- -0.21)^***^	60.0
Women									
Education	Total Indirect	-0.37 (-0.54- -0.20)^***^ -0.19 (-0.24- -0.13)^***^	51.4	-0.17 (-0.35-0.002) -0.19 (-0.25- -0.13)^***^	NA^c^	-0.27 (-0.39- -0.15)^***^ -0.10 (-0.13- -0.06)^***^	37.0	-0.31 (-0.48- -0.13)^***^ -0.19 (-0.25- -0.13)^***^	61.3
Occupation	Total Indirect	-0.21 (-0.42- -0.003)^*^ -0.10 (-0.15- -0.06)^***^	47.6	-0.08 (-0.30-0.14) -0.10 (-0.14- -0.05)^***^	NA^c^	-0.19 (-0.34- -0.05)^**^ -0.06 (-0.08- -0.03)^***^	31.6	-0.22 (-0.44- -0.002)^*^ -0.10 (-0.15- -0.06)^***^	45.5
Wealth	Total Indirect	-0.32 (-0.49- -0.15)^***^ -0.29 (-0.38- -0.21)^***^	90.6	-0.23 (-0.40- -0.05)^**^ -0.29 (-0.37- -0.20)^***^	NA^c^	-0.23 (-0.35- -0.11)^***^ -0.15 (-0.20- -0.09)^***^	65.2	-0.30 (-0.48- -0.12)^***^ -0.30 (-0.39- -0.21)^***^	NA^c^
OSS index	Total Indirect	-0.43 (-0.61- -0.25)^***^ -0.28 (-0.36- -0.20)^***^	65.1	-0.23 (-0.42- -0.04)^*^ -0.28 (-0.37- -0.20)^***^	NA^c^	-0.33 (-0.45- -0.20)^***^ -0.13 (-0.19- -0.08)^***^	39.4	-0.39 (-0.58- -0.20)^***^ -0.28 (-0.37- -0.20)^***^	71.8

^a^Adjusted for age, residence, marital status, and gender (only in the total sample). ^b^% The mediated association was computed as the ratio of the indirect association to the total association. ^c^Full mediation.

^*^Sig. p < 0.05.

^**^Sig. p < 0.01.

^***^Sig. p < 0.001.

NA, not applicable.

## 4 Discussion

The present population-based study showed that the associations between OSS indicators and mental health status were partially to fully mediated through SSS. The most significant mediating role of the SSS was observed in the relationship between wealth and psychological distress.

Previous research has increasingly revealed that OSS has a dose-response association with the development of mental illnesses (37). A recent meta-analysis encompassing 357 studies with a minimum of 2,350,000 participants demonstrated significant associations between objective SES, defined by income and educational attainment, and SWB, which includes measures of happiness and life satisfaction (38). Furthermore, a multi-cohort study of at least 100,000 people found that regional deprivation, education, and occupation status were associated with mental health problems (39). Moreover, a population-based survey conducted in Tehran, involving a sample size of 31,500 participants, revealed that low OSS, as indicated by education and wealth, was significantly associated with poor mental health, as assessed by the GHQ-28 (6). Objective SES significantly influenced the four subscales of the GHQ, encompassing somatic symptoms, anxiety and insomnia, social dysfunction, and depression (6). Stress theory offers a framework for elucidating the mechanisms that link OSS to SWB, emphasizing the role of coping resources and stressors; individuals with higher OSS generally encounter a reduced frequency of stressful and uncontrollable life events and possess greater access to social resources, which attenuate the effects of adverse experiences and improve SWB (17). Moreover, a possible mechanism that has received little attention is the psychosocial roots of health disparities, as there is evidence that the value of objective SES is partly rooted in their perceptions (8).

The cognitive average of OSS factors seems to form subjective SES (9, 15). The OSS-SSS association can be elucidated through the lens of the mindsponge theory. Within this framework, information—such as the information about OSS—undergoes a multi-filtering processing mechanism akin to the absorption capabilities of a sponge. This information processing mechanism is based on trust evaluator as well as subjective cost-benefit judgment. Furthermore, this mechanism depends on a set of core values. The outputs of this cognitive processing include behaviors, ideas, emotions, and thoughts (i.e., SSS) (16). Furthermore, our findings align with social comparison theory, which proposes that achievements, such as OSS, serve as a source of information for the social comparison process (SSS) (40). Previous investigations have demonstrated significant correlations between education, job, wealth, and a composite index of these factors, with the SSS scale (7, 14, 15).

Subjective SES is also affected by individuals’ psychological traits and has a substantial impact on mental health, after adjusting for objective SES (15). SSS has been associated with adverse health consequences across various populations (8). A study analyzing data from 20 surveys in 18 countries, with a sample size of at least 56,000, found graded inverse associations between SSS and 16 mental disorders, with odds ratios ranging from 1.4 to 4.9, after adjusting for OSS factors (10). Moreover, a cross-sectional study derived from the Finnish School Health Promotion Survey, which included a sample of adolescents (N=2,300), indicated that psychological distress, as assessed by the GHQ, was significantly influenced by SSS (boys, OR=5.9; girls, OR=2.5) (41). Additionally, a recent cross-sectional study among 1,000 adolescents and young adults in Ghanaian schools revealed the relationship between SSS and SWB through monetary resources and sense of coherence (13). Nevertheless, a study in Sweden involving around 5,000 adults, after controlling for OSS indicators, did not discover a significant association between SSS and depressive symptoms (12). Besides, the researchers suggest that gender may influence the relationship between SSS and mental disorders (7, 22). The perception of oneself as being of lower social status (SSS) is a chronic stressor that has the potential to modify neuroendocrine function and result in mental illness (42). The social rank theory proposes that an individual’s lower social status than others can lead to a sense of inadequacy and difficulty, ultimately resulting in depression (12). The relative position a person holds in society can significantly influence their actions, thoughts, and views toward the world (43). Subsequently, unfavorable social comparison is associated with negative emotional outcomes such as feelings of hopelessness, worry about the future, and problems with attention (44).

Objective and subjective SES are interrelated and have distinct impacts on health (45). Consequently, a potential association between OSS and mental health through SSS is likely. Nonetheless, the extant literature examining this indirect influence on mental health is constrained to the domain of depression. Our study’s findings are consistent with a cross-sectional study that revealed that SSS predominantly mediated the connection between wealth and depression, and partially mediated the relationships between education and occupational class with depression (14). A recent national health survey also found that there is a significant indirect association between OSS (a composite index created by education, job, and income) and depressive symptoms, as mediated through SSS. The association between income and depressive symptoms declines and nearly disappears when SSS is controlled (7). The US national longitudinal study of adolescent to adult health demonstrated that SSS mediated 27% of the connection between SES and depressive symptoms, 51% of the link between SES and suicidal thoughts, and 37% of the association between SES and suicide attempts on average. The study utilized a composite index based on individual and household income, assets, education, and job prestige to assess SES (15). Furthermore, a cross-sectional study involving 4,400 employees from TUMS reported that the percentages mediated by SSS in the relationships between wealth-mental health, education-mental health, social class-mental health, and OSS-mental health were 42%, 36%, 29%, and 28%, respectively (46). In this research, mental health was assessed using the Depression, Anxiety, and Stress Scale (46). Moreover, a meta-analysis study demonstrated the indirect association of education and income on SWB, specifically happiness and life satisfaction, through subjective SES, particularly by utilizing the MacArthur ladder measure (38). Additionally, a cross-sectional study conducted using data from the Chinese General Social Survey, which included a sample of 1,900 adults, demonstrated that subjective class identity serves as a significant mediating factor in the relationship between OSS—a composite measure derived from educational attainment and income—and SWB (17). Subjective class identity refers to an individual’s self-perception regarding their position within the social class hierarchy (17).

Our findings demonstrated the indirect influences of objective SES on GHQ scales, supported by the scholarly literature. A representative sample of Spanish adolescents (N=15,300) revealed the significant indirect associations of objective SES on psychosomatic symptoms, mediated by SSS (47). Low subjective SES may contribute to the development of psychosomatic complaints via an unhealthy lifestyle (47). Moreover, population-based surveys involving a sample of 13,700 adults in the United States indicated that individuals experiencing residential instability, lower income, and diminished SSS are at a heightened risk for anxiety disorder (48). People from low-wealth backgrounds, who possess lower perceptions of their social standing, encounter elevated financial stress, which correlates with increased levels of anxiety (12). Furthermore, a review study encompassing 336 investigations has revealed individuals with low educational attainment, occupational class, and income levels are more susceptible to developing insomnia, with subjective SES being the most substantial predictor (49). Individuals with lower perceived social status experience elevated stress levels, which can ultimately lead to the development of insomnia (49). Sleep health has been widely recognized as a crucial determinant of mental health (49). The social function scale of the GHQ-28 is designed to assess performance satisfaction and fulfillment, sense of participation, and enjoyment derived from daily activities (29). A survey with 17,200 residents of China reported that subjective SES, serving as a significant complement to objective SES, predicted life satisfaction within the framework of social capital (50). Life satisfaction is defined as the cognitive assessment of the extent to which personal expectations and standards are met, coupled with the emotional experience of contentment and pleasure derived from one’s life (50).

Our findings indicated that the total and indirect associations of each objective SES factor with mental health status did not exhibit a statistically significant difference between men and women participants. Moreover, the mediating roles of SSS in the relationships between OSS factors and mental health status are almost similar across both genders. Several studies have shown that mental health is substantially influenced by SSS (10, 13, 41). Furthermore, a cross-sectional study derived from a nationally representative sample of over 5,000 American adults indicated that gender did not exert a significant moderating role in the relationship between SSS and depressive symptoms (51). Hence, the comparable SSS percentages mediated in both genders may be a result of the comparable association between SSS and mental health status in both women and men populations.

### 4.1 Strengths and limitations

Our study possesses notable strengths. First, we utilized national data to provide more representative data and greater statistical power. Second, previous research assessed objective SES using a general index, neglecting to examine the individual of its components—education, occupation, and wealth or income—to mental health in the hypothetical causal chain (7, 15). We employed a broad range of OSS indicators. Education and occupation are identified as the primary dimensions of OSS, each possessing unique characteristics and underlying mechanisms in mental health (7). In addition, we used wealth as a metric for material inequalities that confer significant advantages. This is because wealth represents the mastery of financial resources, reflecting accumulated advantages and future economic predictions (14). Wealth serves as a more stable indicator of economic status than income, mitigating the effects of temporary income loss or low income. Therefore, it may have a more profound impact on health than income (52). Besides, the integration of OSS indicators into a composite index yields more comprehensive assessments of the social gradient in mental health issues (7). Third, in contrast to earlier studies that focused on depressive symptoms (7, 14, 15), this research utilized the GHQ-28 to assess mental health status more comprehensively. Additionally, we incorporated the four dimensions of the GHQ-28 including somatic symptoms, anxiety and insomnia, social dysfunction, and depression into the mediation analyses.

Our cross-sectional data has limitations in illustrating causal inferences. The plausibility of the consequence of depression on SSS is supported by the notion that individuals with depressed mood tend to have a poorer appraisal of their SSS compared to those without depressed mood (7). Longitudinal data analysis revealed that the associations between SSS and health can be attributed to effects operating in both directions (8). Nevertheless, according to longitudinal data from Taiwan, a higher level of SSS is negatively associated with the risk of depression, even after adjusting for baseline depressive symptoms (53). Another constraint arises from the reliance on self-reported household assets for wealth assessment, possibly introducing social desirability bias. This bias can distort reported wealth levels as individuals may manipulate asset disclosure to portray a more positive financial standing (54). However, measuring individuals’ wealth and assets presents complexities, particularly in developing nations lacking a dedicated registration framework for assessing individuals’ asset holdings. Additionally, our study did not investigate the possible moderating role of subjective SES on the relationship between objective SES and mental health status. The data used in this study were collected in 2011, which may limit the relevance of our findings to the current socioeconomic context. Social and economic conditions in Iran have likely changed over the past decade, and these changes could affect the associations between OSS, SSS, and mental health outcomes. Future research should consider using more recent data to validate and extend our findings in light of the evolving socioeconomic landscape.

### 4.2 Implications and future research

Our findings suggest that subjective SES can explain part of the relationship between objective SES and mental health status, highlighting its importance in understanding mental health disparities. Future research could benefit from prioritizing longitudinal studies to further explore the role of subjective SES as both a mediator and moderator in the relationship between objective SES and mental health outcomes. Additionally, it may be valuable to investigate interventions aimed at enhancing subjective SES among individuals with lower objective SES, to assess potential improvements in mental health.

In clinical practice, it might be beneficial for clinicians and psychotherapists to consider incorporating assessments of subjective social status alongside traditional objective SES measures when evaluating clients’ mental health. Policymakers and decision-makers could also consider the relevance of subjective social status when formulating and implementing mental health policies and programs.

Community organizations could explore the potential of designing and implementing programs that promote social engagement and the development of support networks, which may enhance individuals’ subjective SES. By fostering environments where individuals feel respected and valued, these initiatives could contribute to narrowing the gap between objective and subjective SES, which may be associated with improved mental health outcomes.

### 4.3 Conclusions

This study, utilizing a nationally representative sample from the IranMHS, provides important insights into the complex relationships between objective socioeconomic status (OSS), subjective social status (SSS), and psychological distress as measured by the GHQ-28. Our findings demonstrate that SSS plays significant mediating roles in the associations between OSS indicators—such as education, occupation, and wealth—with mental health status. Notably, the mediating role of SSS was found to be substantial, particularly in the relationship between wealth and psychological distress.

The study underscores the importance of considering both objective and subjective measures of SES in understanding mental health disparities. These results suggest that enhancing individuals’ subjective social status, especially among those with lower objective socioeconomic status, could be a valuable strategy for mitigating psychological distress and improving mental health outcomes.

Future research should explore these relationships further, particularly through longitudinal studies, to better understand the causal pathways involved. Additionally, the findings highlight the potential value of integrating SSS assessments in clinical practice and policy-making, as addressing the subjective perceptions of social status may contribute to more effective mental health interventions.

## Data Availability

The raw data supporting the conclusions of this article will be made available by the authors, without undue reservation.
