# Supplementary material for: The mediating role of subjective social status in the association between objective socioeconomic status and mental health status: evidence from Iranian national data
Source: Front Psychiatry. 2024 Sep 30;15:1427993. doi: 10.3389/fpsyt.2024.1427993 (PMC11471600; doi:10.3389/fpsyt.2024.1427993)
Supplement: Supplementary file 1 [file Table1.docx]

Supplementary Material

# 1 Supplementary Tables

**Supplementary Table S1.** Factor scores of household assets and amenities based on principal component analysis (PCA)

| Household asset or amenity | Proportion owning (%) | Factor score |
| --- | --- | --- |
| Kitchen | 90.2 | 0.2715 |
| Private bath | 89.0 | 0.3013 |
| Private toilet | 92.7 | 0.2421 |
| Refrigerator | 99.0 | 0.0783 |
| Freestanding freezer | 67.2 | -0.0954 |
| Black and White TV | 3.2 | -0.0295 |
| Color TV | 89.2 | -0.125 |
| LCD/LED TV | 15.9 | 0.2678 |
| Mobile phone | 90.2 | 0.1721 |
| Washing machine | 67.5 | 0.3146 |
| Dishwasher | 4.5 | 0.187 |
| Microwave | 13.3 | 0.2409 |
| Vacuum cleaner | 79.1 | 0.3082 |
| Computer/ laptop | 33.6 | 0.3651 |
| Internet access | 21.0 | 0.3351 |
| Motocycle | 34.2 | -0.0337 |
| Car | 44.6 | 0.2919 |
| House | 68.0 | 0.1037 |
| Villa or garden property | 7.3 | 0.119 |

**Supplementary Table S2.** Wealth groups based on wealth score ranges derived from PCA

| Wealth group | Range | |
| --- | --- | --- |
|  | Lower limit | Upper limit |
| 1 | -5.920 | -1.725 |
| 2 | -1.724 | -0.691 |
| 3 | -0.690 | -0.079 |
| 4 | -0.078 | 0.694 |
| 5 | 0.695 | 1.901 |
| 6 | 1.902 | 5.330 |
